# Spotlight on zebrafish: the next wave of translational research

**DOI:** 10.1242/dmm.039370

**Published:** 2019-03-07

**Authors:** E. Elizabeth Patton, David M. Tobin

**Affiliations:** 1 MRC Human Genetics Unit, MRC Institute of Genetics and Molecular Medicine, University of Edinburgh, Western General Hospital, Crewe Road South, Edinburgh EH4 2XU, UK; 2 Departments of Molecular Genetics and Microbiology, and Immunology, Box 3020, Duke University School of Medicine, Durham, NC 27710, USA

**Keywords:** Disease models, Genome editing, Zebrafish

## Abstract

Five years after the launch of the Disease Models & Mechanisms (DMM) Special Issue on zebrafish as a disease model, the field has progressed significantly. Zebrafish have been used to precisely model human genetic variants, to unpick the mechanisms of metabolic and other diseases, to study infection, inflammation and cancer, and to develop and test new therapeutic approaches. In this Editorial, we highlight recent research published in DMM that uses zebrafish to develop new experimental tools and to provide new insight into disease mechanism and therapy. The broad spectrum of subjects and approaches covered in these articles underscores the versatility of zebrafish in translational research. Further, it highlights the zebrafish community's ethos of creativity and collaboration in translating basic biological research into clinically relevant advances affecting how we understand and treat human disease.

## Abstract (precis)

**Summary:** Zebrafish are a highly versatile and relevant organism for human disease modelling. This Editorial highlights the recent zebrafish research published in DMM.

## Introduction

It is a great pleasure to introduce the next wave of zebrafish papers at Disease Models & Mechanisms (DMM). In 2014, DMM launched a Special Issue to highlight zebrafish in translational science (Patton et al., 2014). The use of zebrafish as a disease model has increased dramatically in the past 5 years. To mark this progress, we are delighted to celebrate some of the recent papers published in DMM that use zebrafish to model disease and provide new insight into disease mechanism and therapy.

Zebrafish continue to be a ‘model’ model organism for disease and translational research. Zebrafish are vertebrates and share genes, development and disease states with humans. Their optical transparency in early embryogenesis enables exquisite imaging of developmental processes, response to drugs, regeneration and infection. Zebrafish adults share much of the physiology and anatomy with humans and other vertebrates, and many of the disease processes of the adult zebrafish directly model clinical disease and outcome. Notably, an emerging theme in this collection is the combination of using zebrafish with other model systems, such as the mouse, to fully explore disease mechanism or therapy. The growing zebrafish disease models community, the quality of the DMM scientific papers and reviewer reports, and that zebrafish papers receive some of the highest citation numbers at DMM provide further evidence of the importance of zebrafish as a disease model.

## Modelling human genetics

One of the most important recent technological advances for zebrafish disease modelling has been in precision genome editing. CRISPR-Cas9 has enabled the rapid and efficient generation of genetic mutations in zebrafish, and, importantly, this can be used to generate site-specific mutations in a tissue-specific manner. An example of the importance of this technology is demonstrated by work from the Bakkers laboratory, in which they use CRISPR-Cas9 with short template donor oligonucleotides to edit mutations associated with Cantú syndrome into zebrafish orthologous genes, and demonstrate that the fish develop cardiovascular disorders that are clinical hallmarks of this syndrome (Tessadori et al., 2018).

Predicting disease-causing gene variants can be difficult because many human disorders are caused by complex genetics involving combinations of low-penetrant genetic variants. Maves and colleagues demonstrate the power of gene editing in zebrafish for modelling complex genetics in an elegant study that generates zebrafish with a disease-associated *pbx3* single-nucleotide variant of unknown significance that is associated with congenital heart defects (Farr et al., 2018). They then show that the *pbx3* variant enhances the cardiac morphogenesis defects caused by mutations in known cardiac factors. In this way, precision gene editing in zebrafish can provide a powerful platform for new gene–gene (and even gene–environment) interactions.

CRISPR-Cas9 enables the rapid testing of candidate disease genes identified through human genetics. The Sumanas laboratory uses CRISPR-Cas9 to generate mutations in the zebrafish gene encoding Col22A1 collagen, a gene in which they find an E736D mutation in patients affected by intracranial aneurysms. In zebrafish, they show that *col22a1* mutants develop intracranial haemorrhages, and that the E736D mutant may interfere with COL22A1 secretion (Ton et al., 2018). Together, these results point to an important function for COL22A1 in vascular integrity and suggest that mutations in *COL22A1* may contribute to the development of intracranial aneurysms.

The accessibility of zebrafish embryos means that zebrafish can inform about the earliest pathogenic mechanism or marker in genetic models of human disease. This is the case for the new CRISPR-Cas9 zebrafish and mouse models of kyphoscoliosis peptidase (*kp*)-deficient myopathies described by Blanco and colleagues, which show increased and dysregulated expression of autophagy factors that appear to be early hallmarks of the disease (Jokl et al., 2018). Similarly, the Traver laboratory used zebrafish genetics to explore the potential for Mecp2 to regulate the immune response, and found that the pro-inflammatory cytokine TNFα was downregulated in *mecp2* mutant animals and unresponsive to inflammatory stimuli (van der Vaart et al., 2017). Mecp2 is mutated in Rett syndrome, a severe neurological disorder, and the downregulation of TNFα is, at present, the earliest developmental phenotype described for Rett syndrome models. These early markers of disease found in *kp* mutant zebrafish and mice, and in *mecp2*-deficient zebrafish, may have important implications for understanding disease progression and early treatment interventions.

## Metabolic disease in zebrafish

A fascinating area of rapid expansion in zebrafish disease models is in obesity and metabolic diseases, which leave patients at risk for dyslipidaemia, type II diabetes and fatty liver disease. In this collection, we highlight some of the most innovative models in this area. For fat metabolism, Minchin and Rawls contribute a landmark paper that identifies, quantifies and enables visualization of zebrafish adipose tissue dynamics (Minchin and Rawls, 2017), and that has already been instrumental in the validation of new obesity disease variants (van der Klaauw et al., 2019). Further, the Farber laboratory uses zebrafish to visualize Caveolin 1 mediating transport between the enterocytes and the submucosa, and their observations, combined with mouse genetics, point to Caveolin 1 as a potential therapeutic target to lower circulating fat and cholesterol (Otis et al., 2017).

For modelling diabetes, zebrafish blood sugar levels can be altered experimentally by adding glucose to the water. Using these approaches, the Connaughton laboratory shows that prolonged hyperglycaemia for adult zebrafish triggers an inflammatory response in the retina, leading to functional deficits in cone cells that could ultimately be important to understand how diabetes can lead to blindness (Tanvir et al., 2018). Hyperglycaemia is a further risk factor for diabetic cardiomyopathy, and the Chen laboratory uses adult zebrafish to model hyperglycaemia-induced cardiomyopathy and identify the Nkx2.5–Calr–p53 signalling pathway as a key regulator of this process (Sun et al., 2017).

While metabolic diseases are most often attributed to genes and lifestyle, Sadler and colleagues provide stunning new evidence in zebrafish to show that chronic exposure to inorganic arsenic can cause fatty liver disease, and, further, that combining subtoxic concentrations of arsenic with ethanol is sufficient to promote the disease through increased oxidative and endoplasmic reticulum stress responses (Bambino et al., 2018). This model supports epidemiological evidence that environmental toxicants can contribute to metabolic disease; for example, high levels of arsenic are found in drinking water in regions of Bangladesh and India with high levels of fatty liver disease but without lifestyle risk factors.

The burden of metabolic disease on the individual and society requires new drugs and targets. Current metabolic disease treatment includes peroxisome-proliferator-activated-receptor (PPAR)-targeted drugs, but these are associated with unwanted toxic effects. The Krause laboratory has generated transgenic zebrafish models for PPAR target gene expression, which can be used to screen for new PPAR partial agonists that act in a tissue-specific way to reduce toxicity. Using this approach, they identify a structural analogue of coenzyme Q_10_ (idebenone) that effectively reversed fatty liver disease in mouse models of type 2 diabetes (Tiefenbach et al., 2018). It will be exciting to follow how these and other new drug leads alter disease in the above zebrafish models of metabolic disease, and their progression toward the clinic.

## Inflammation and infection models

The zebrafish has emerged as a particularly useful system for probing the *in vivo* cellular and molecular architecture of inflammation and infection. Sterile models of inflammation in zebrafish have highlighted aspects of neutrophil biology that are conserved among vertebrates. Two papers from the Deng laboratory provide new insights into how inflammation can be regulated and manipulated. The Rho GTPase Rac2 plays an essential role in macrophage and neutrophil motility, and contributes to other neutrophil functions. After identifying a microRNA (miRNA) that binds the *rac2* 3′ UTR, the authors show that the neutrophil-specific expression of the miRNA could be used to directly modulate systemic inflammation (Hsu et al., 2017). In a second paper, the Deng laboratory develops a system for the first neutrophil-specific gene knockouts in zebrafish using cell-specific CRISPR/Cas9 expression (Zhou et al., 2018). Using this system, they go on to demonstrate a cell-autonomous requirement for specific mitochondrial components in neutrophil function. This ability to perform cell-type-specific knockouts and knockdowns more easily in zebrafish (Ablain and Zon, 2016) will be particularly important as the field moves from whole-animal mutants and knockdowns to conditional alleles and cell-specific disruptions.

Zebrafish models have entered new territory in our understanding of infectious diseases. A review from the Mostowy laboratory highlights how the intricate cell biology of infection can be visualized and probed live in zebrafish, focusing on *Shigella*, and how many known, essential features of *Shigella* pathogenesis can be seen during zebrafish larval infections (Duggan and Mostowy, 2018). Even unnatural pathogen–host pairings in the zebrafish can provide new insight into fundamental features of pathogens and their interactions with a vertebrate host, as properties of that pathogen and cell-type-specific biology and interactions are conserved. One particularly elegant study from the Levraud laboratory highlighted here describes intravital imaging of viral entry into the central nervous system (CNS), comparing the trajectories of fluorescent Chikungunya virus and Sindbis virus (Passoni et al., 2017). Crossing of the blood–brain barrier and entry into the CNS is a dangerous complication of infection with a number of pathogens – viral, fungal and bacterial. The zebrafish should provide a useful platform for understanding each of these pathogen classes and testing potential mechanisms for CNS invasion.

*Mycobacterium marinum*, a close relative of *Mycobacterium tuberculosis*, is a natural pathogen of fish. Its study in zebrafish has provided mechanistic insights that have translated into an understanding of conserved biology of tuberculosis in humans. Two papers in this collection highlight the advantages of zebrafish as a discovery platform for identifying potential therapeutic avenues. Using an adult model of infection, the Parikka laboratory demonstrates dramatically increased sterilizing immunity to mycobacterial infection after immune priming with heat-killed *Listeria monocytogenes* (Luukinen et al., 2018). The effect may be mediated through a process called trained immunity, or innate immune memory, an exciting and active new area of study in mammals and humans. A paper from the Rämet laboratory uses the adult infection model to screen 15 different mycobacterial antigens as post-exposure vaccines for mycobacterial infection, identifying two new mycobacterial antigens with substantial efficacy (Myllymäki et al., 2017).

## Cancer models in zebrafish

Zebrafish research has generated powerful cancer models, and this collection highlights two models of brain cancer that identify new targets for these diseases. The Mione laboratory presents evidence that somatic RAS mutations expressed in the brain can promote both heterotopia and invasive tumours, suggesting that brain tumours may originate from a pre-malignant developmental lesion (Mayrhofer et al., 2017). Importantly, the authors discover that the zebrafish tumours are enriched for a mesenchymal glioblastoma subtype gene expression signature with a strong YAP component, and then identify a simple YAP eight-gene signature that can distinguish between human mesenchymal glioblastoma and low-grade glioma. This is an important example of how zebrafish models can directly inform about human cancer. McGrail and colleagues use transcriptomic approaches to identify a new epigenetic mechanism for mutant RB1 in primitive neuroectodermal-like brain tumours. They then explore the mechanism of chromatin remodellers in neural stem and progenitor cells in development using an *in vivo* assay that involves combining somatic CRISPR targeting with live imaging of the histone-H2A.F/Z-GFP fusion protein in developing larval brain (Schultz et al., 2018). These findings may have important implications for new potential targets in RB1 mutant brain cancer.

An elegant forward genetic screen from the Bowman laboratory identified spliceosome components as protective against ionizing-radiation-induced apoptosis in embryonic neurons. The formation of R-loops in these mutants led to DNA double-strand breaks and apoptosis, making neuronal tissues more radiosensitive, and suggesting that these pathways may be targeted or modified therapeutically in certain cancers (Sorrells et al., 2018).

Understanding how immune cells interact with cancer cells is critical for immune-based therapies, and zebrafish provide an ideal model system to address these questions. To address how newly transformed cancer cells interact with the immune system, the Sauka-Spengler laboratory has developed an *in vivo* myeloid-specific biotagging system to profile the active transcriptome of the immune response to the earliest stages of melanoma (Kenyon et al., 2018). Interactions with macrophages can have profound implications for the tumour, as shown by a contribution from the Astin laboratory. They leverage the power of live imaging in zebrafish to show how cancer xenografts can induce vascularization and recruit immune cells, and show how macrophages can augment Vegfa-driven tumour angiogenesis (Britto et al., 2018).

In addition to interactions with the immune system, cancers can disrupt host physiology, a phenomenon exemplified by cachexia in cancer patients. In a highly original paper by Kawaoka and colleagues, the authors developed a model for Kras-driven intestinal cancer and studied the systemic effects of the tumour on otherwise healthy tissues. They discovered that, despite the benign state of the tumour, it induced systemic phenotypes that included liver inflammation, hepatomegaly and overall growth delay (Enya et al., 2018). The mechanism for the liver inflammation was found to be mediated via the host *cyp7a1* gene that regulates hepatic cholesterol–BA metabolism. It will be exciting to follow the progression of this new field of whole-zebrafish experiments to discover physiologically important tumour–organ interactions.

## Drug discovery and development

By the fifth day of development, zebrafish have organs and tissues that are functional and they display stereotypical behaviours. This remarkable rapid development, coupled with their small size and transparency, make zebrafish ideal for whole-organism chemical biology and drug discovery.

This collection highlights the importance of zebrafish phenotype-based drug screens for human disease. Porter and colleagues develop a new model for Niemann–Pick disease, a severe metabolic disorder of sphingolipids characterized by an accumulation of cholesterol and glycolipids in late endosomes/lysosomes, and demonstrate how it can be used as a robust platform for *in vivo* screening of therapeutic compounds (Tseng et al., 2018). The Xiong laboratory identifies miconazole as a haemorrhagic suppressor through a small-molecule screen in zebrafish (Yang et al., 2017). This is important because although haemorrhagic strokes are less common than other types of strokes, they are responsible for over 40% of all stroke deaths and there are few therapeutic interventions available. Ninov and colleagues develop a new diabetes model in the zebrafish embryo based on pancreatic β-cell inflammation, and show that the natural product wedelolactone can protect against diabetes in zebrafish, and, notably, also protects mouse and human β-cells from inflammation (Delgadillo-Silva et al., 2019).

One of the challenges in the drug-development field is assessing the pharmacokinetics of a drug in the context of the whole animal. In a fascinating study, Wang and colleagues test a large panel of fluorescent molecules in zebrafish and find 15 molecules with tissue-specific distributions (Yao et al., 2017). These included new fluorescent bone dyes that proved effective in live zebrafish and in postnatal mice, and enabled the *in vivo* visual tracing of bone development in fish and mammals without impacting upon bone properties such as density, length or mass. The tissue-specific distribution of molecules is important for disease treatment. The authors identified two florescent chemotherapeutic drugs, epirubicin and doxorubicin, and found that the liver-specific distribution of epirubicin was associated with a greater anti-proliferative effect in a model of *kras^G12V^*-driven hepatic hyperplasia. These studies provide a state-of-the-art approach to understanding how to minimize off-target toxicities and may have important implications for drug efficacy.

## New methods and tools

A major limiting factor for studying diseases in zebrafish is the development of appropriate tools. In this collection, the Xu and Fatkin laboratories present two new resource methods to assess cardiac function in adult zebrafish that can detect and reveal disease processes (Wang et al., 2017; Zhang et al., 2018). In cancer, the White laboratory presents an exciting new method for generating adult zebrafish cancer models called transgene electroporation in adult zebrafish (TEAZ), which helps to better model somatic gene mutations in a spatially defined tissue (Callahan et al., 2018). The Meyer laboratory achieved conditional, genetically encoded, cell-specific ablation of exocrine cells in zebrafish using transgenic lines expressing the human diphtheria toxin receptor or, alternatively, a membrane-localized Caspase 8-FKBP fusion, in which apoptosis could be pharmacologically induced (Schmitner et al., 2017). In addition to robust, conditional ablation of exocrine tissue, these tools allow a detailed examination of progenitor populations during regenerative processes.

Creative new approaches to integrate mammalian reagents and tools with a zebrafish platform are exemplified by work from the Allende laboratory. Transplantation of murine bone marrow cells into the zebrafish blastula generates chimeric embryos in which murine hematopoietic cells home to zebrafish hematopoietic tissues, circulate and can respond to bacterial infection (Parada-Kusz et al., 2018).

New technology requires optimization and characterization methods, and, in this collection, the Berman laboratory provides a method to recover and validate frameshift mutations (Prykhozhij et al., 2017), while the Willaert laboratory provides methods to improve error-free rates of DNA repair (Boel et al., 2018). Capturing the excitement of gene editing in zebrafish, an editorial by the Berman laboratory provides an up-to-date view of the state of the art for genome editing in zebrafish (Prykhozhij and Berman, 2018).

## Conclusions

The broad spectrum of subjects and approaches covered in this collection highlights the versatility of zebrafish in translational research. New technologies for precise genetic manipulations have now enabled the examination of specific genetic variants and specific cell types within the context of a whole, live organism. Exploration of adult biology has expanded the range of topics that can be examined and diseases that can be modelled. Zebrafish research has a strong track record of uncovering conserved biological pathways, mechanisms and principles that provide new insight into human biology and disease. The zebrafish community has a strong clinical scientist base, and clinical findings and observations inspire new zebrafish studies to uncover their underlying biological basis in mechanistic detail. This collection reflects the zebrafish community's ethos of creativity and collaboration, as basic research findings, observations and insights contribute to our understanding of human disease.
